# Correlation of increased serum leucine-rich α2-glycoprotein levels with disease prognosis, progression, and activity of interstitial pneumonia in patients with dermatomyositis: A retrospective study

**DOI:** 10.1371/journal.pone.0234090

**Published:** 2020-06-01

**Authors:** Takaaki Ishida, Takuya Kotani, Satoshi Serada, Minoru Fujimoto, Tohru Takeuchi, Shigeki Makino, Tetsuji Naka

**Affiliations:** 1 Department of Internal Medicine (IV), Osaka Medical College, Takatsuki, Osaka, Japan; 2 Center for Intractable Immune Disease, Kochi Medical School, Kochi University, Nankoku, Kochi, Japan; Nippon Medical School, JAPAN

## Abstract

**Objective:**

To investigate whether leucine-rich α2-glycoprotein (LRG) can be a biomarker for the disease activity, progression, and prognosis of interstitial pneumonia (IP) in patients with dermatomyositis (DM).

**Methods:**

Correlations between the clinical findings and serum LRG levels were investigated in 46 patients with DM-IP (33 with acute/subacute IP [A/SIP] and 13 patients with chronic IP [CIP], including 10 fatal cases of IP).

**Results:**

The median serum LRG level of 18.4 (14.6–25.2) μg/mL in DM-IP patients was higher than that in healthy control subjects. The median levels of serum LRG at baseline and at 2 and 4 weeks after the initiation of treatment in the patients who died were significantly higher than those in the surviving patients (*P* = 0.026, 0.029, and 0.008, respectively). The median level of serum LRG in the DM-A/SIP patients was significantly higher than that in the DM-CIP patients (*P* = 0.0004), and that in the anti-MDA5-Ab-positive group was slightly higher than that in the anti-ARS-Ab-positive group. The serum LRG levels correlated significantly with the serum levels of LDH, C-reactive protein, ferritin, AaDO_2_, %DLco, and total ground-glass opacity score. The survival rate after 24 weeks in patients with an initial LRG level ≥ 17.6 μg/mL (survival rate: 40%) was significantly lower than that in patients with an initial LRG level < 17.6 μg/mL (100%) (*P* = 0.0009).

**Conclusion:**

The serum LRG level may be a promising marker of disease activity, progression, and prognosis in patients with DM-IP.

## Introduction

Dermatomyositis (DM) is frequently complicated by interstitial pneumonia (IP), which is associated with increased morbidity and mortality [1, 2]. DM-IP is classified as being either acute/subacute IP (A/SIP) or chronic IP (CIP), and A/SIP type progresses within 3 months. Anti-aminoacyl tRNA synthetase (ARS) antibody (Ab) and anti-melanoma differentiation-associated gene (MDA) 5 Ab are autoantibodies associated with DM-IP. Patients with advanced disease and are positive for anti-ARS Ab undergo immunosuppressive therapy with corticosteroids, calcineurin inhibitor, and/or intravenous pulse cyclophosphamide (IVCY). Although most patients respond well to immunosuppressive therapy, some are refractory or experience recurrence [3, 4]. However, although anti-MDA5 Ab-positive DM-IP patients are immediately placed on aggressive immunosuppressive therapy, about half of these patients are resistant to the therapy and will die from respiratory failure within a few months [5, 6]. Thus, when determining the appropriate treatment, it is clinically important to discover factors predictive of the progression and prognosis of DM-IP.

Leucine-rich α2-glycoprotein (LRG) is a 50-kDa glycoprotein containing repetitive sequences with a leucine-rich motif [7, 8] and is expressed by hepatocytes and neutrophils [9, 10]. Serum LRG is reported as a biomarker of inflammatory diseases such as rheumatoid arthritis, adult-onset Still’s disease, Kawasaki disease, and inflammatory bowel disease [11–15]; however, its association with the pathogenesis of DM-IP has not been clarified. In this study, we compared serum LRG levels of DM-IP patients and healthy control subjects (HC) and investigated treatment-induced changes in the serum LRG levels of DM-IP patients. The correlations between the serum LRG level and other clinical parameters were examined to evaluate the usefulness of the serum LRG level as a marker of the disease activity, progression, and prognosis of DM-IP.

## Materials and methods

### Patients

This retrospective study included patients with DM admitted to Osaka Medical College Hospital between October 2011 and November 2017. DM and clinically amyopathic DM (CADM) were diagnosed according to the criteria of Bohan and Peter [16, 17] and Sontheimer and Gerami et al [18, 19]. Patients with an overlapping syndrome such as other connective tissue disease or malignancy were excluded. IP was diagnosed by chest high-resolution computed tomography (HRCT). A/SIP was defined according to American Thoracic Society (ATS) and the European Respiratory Society (ERS) consensus statement as follows: IP with a respiratory condition, laboratory findings, arterial blood gas findings, chest HRCT scans, and pulmonary function test findings rapidly progressing within a period of days to 3 months after disease onset. Patients with CIP are those who did not meet the definition of A/SIP [20]. The clinical and laboratory findings, and treatment contents of the patients were obtained from medical records on hospital admission. This study was approved by the ethical committee of Osaka Medical College (No. 1785) and complied with the guidelines of the Declaration of Helsinki. Written informed consent was obtained from each patient. For patients who had died, we contacted their home via postal mail to determine whether their family members would allow participation in this study.

### Measurement of laboratory parameters

The laboratory test items evaluated were creatine kinase (CK), lactic acid dehydrogenase (LDH), C-reactive protein (CRP), Krebs von der Lungen-6 (KL-6), and ferritin. Blood serum samples were collected on admission (baseline measurement) and at 2, 4, and 8 weeks after treatment initiation. The serum samples were stored at −30°C until measurement of the following autoantibodies. Anti-MDA5 Ab and anti-aminoacyl-tRNA synthetase (ARS) Ab were examined by enzyme-linked immunosorbent assay (ELISA) (MESACUP^™^ anti-MDA5 kit; MBL, Nagoya, Japan) and blot assay (Myositis Profile Euroline Blot test kit; EUROIMMUN, Lübeck, Germany), respectively. Serum LRG levels were also analyzed by ELISA as described previously [21].

### Arterial blood gas analysis and pulmonary function testing

Arterial blood gas analysis, including PaO_2_, PaCO_2_, and alveolar-arterial oxygen difference (AaDO_2_), was performed on admission. Static and dynamic lung volumes were measured by spirometry (SYSTEM21; Minato Medical Science, Osaka, Japan). Vital capacity (VC) was determined by the N2 washout method, and diffusion capacity of the lung for carbon monoxide (DLco) was measured by the single-breath method. The pulmonary function test results are expressed as percentages of the predicted value.

### HRCT scoring

HRCT was performed with a 64-detector row CT Aquilon multiscanner (Toshiba Corporation Medical System, Tokyo, Japan). The slice thickness was 1.0–1.5 mm every 10 mm, with the scan area including the entire lung. All patients underwent chest HRCT prior to treatment, and images were reviewed independently by 3 pulmonologists (TK, TI, and TS) blinded to the clinical information. Inter-observer disagreements were resolved by consensus. Ground-glass opacity (GGO) and fibrosis were both scored to assess HRCT findings, as described previously [22]. The lobes of each patient were scored by the same observers, and the average value was used. The scores obtained were summed as the total CT score.

### Statistical methods

Statistical analysis was performed with the Mann-Whitney U-test to compare median values and Fisher’s exact test to compare frequencies. Wilcoxon’s rank sum test was used to assess the LRG level at the baseline measurement and at 2, 4, and 8 weeks after treatment initiation. We used receiver operating characteristic (ROC) curve analysis to determine the most suitable cut-off level by calculating the Youden index. We applied the Kaplan-Meier method to assess survival curves and the log-rank test to evaluate the significance of differences between the two groups. Correlations were evaluated using Spearman’s correlation coefficients. A *P*-value <0.05 was considered significant. The data were analyzed with JMP software, version 12.0.1 (SAS Institute, Cary, NC, USA).

## Results

### Comparison of clinical and laboratory findings between survivors and non-survivors

Table 1 shows the clinical and laboratory findings and treatments for the 10 patients who died of DM-IP and the 36 survivors. No significant differences were observed in age, sex, number of patients with CADM, or time from the appearance of respiratory symptoms to treatment initiation between the surviving patients and those who died. The percentage with A/SIP was significantly higher in the IP death group (100%) than in the survivor group (64%) (*P* = 0.042). In comparisons of the IP death group versus the survivor group, the anti-MDA5-Ab-positive rate was significantly higher (80% vs. 14%; *P* = 0.0002), the anti-ARS-Ab-positive rate was significantly lower (0% vs. 56%; *P* = 0.0024), and the serum levels of LDH, ferritin, and LRG were significantly higher (*P* = 0.011, 0.0008, and 0.026, respectively). No significant differences were noted in the serum levels of CK, CRP, or KL-6 between the two groups. No significant differences were noted in %VC or %DLco, but the AaDO_2_ and total GGO score were significantly higher in those who died than in the survivors (*P* = 0.0001 and 0.018). There were no differences in the treatment doses of PSL, CSA, or total IVCY, but MPDN pulse therapy and intravenous immunoglobulin were used significantly more frequently in those who died.

**Table 1:** Clinical and laboratory findings of the survivors and dead patients.

Characteristics	Dead (n = 10)	Alive (n = 36)	*P*
Age, years	64.5 (54.3–70.0)	56.0 (47.0–67.8)	0.305
Female, n (%)	5 (50)	28 (78)	0.117
CADM, n (%)	8 (80)	23 (64)	0.460
A/SIP, n (%)	10 (100)	23 (64)	0.042*
Disease duration, months	1.1 (0.5–2.8)	2.0 (0.6–4.8)	0.258
Positive anti-MDA5-Ab, n (%)	8 (80)	5 (14)	0.0002*
Positive anti-ARS-Ab, n (%)	0 (0)	20 (56)	0.0024*
CK, IU/l	213 (66.8–731)	104 (55.8–265)	0.432
LDH, IU/l	428 (333–504)	261 (206–382)	0.011*
CRP, mg/dl	0.91 (0.67–1.74)	0.16 (0.06–2.15)	0.068
KL-6, U/ml	1188 (629–2309)	717 (477–1117)	0.126
Ferritin, ng/ml	1330 (873–1773)	132 (57.1–619)^c^	0.0008*
LRG, μg/ml	23.7 (19.5–26.0)	16.6 (11.3–23.6)	0.026*
AaDO_2_, mmHg	71.0 (44.1–94.9)	20.6 (9.75–33.4)	0.0001*
%VC, %	60.3 (49.8–102.1)^a^	83.3 (76.5–93.4)^d^	0.405
%DLco, %	24.9 (15.4–72.3)^a^	52.4 (39.1–64.0)^d^	0.469
Total GGO score	15.7 (10.6–18.1)	8.2 (6.6–12.8)	0.018*
Total fibrosis score	5.0 (2.6–5.0)	3.3 (2.4–4.6)	0.096
PSL, mg/day	55 (50–65)^b^	50 (35–60)	0.101
CSA, mg/day	238 (219–256)	250 (200–250)^e^	0.710
TAC, mg/day	–	4.5 (4.0–6.8)^f^	-
MPDN pulse, n (%)	7 (70)	4 (11)	0.0006*
IVCY, n (%)	10 (100)	15 (42)	0.0009*
Total IVCY, mg	1700 (1000–3625)	2100 (1000–4000)^e^	0.911
IVIg, n (%)	5 (50)	6 (17)	0.043*

Laboratory markers are presented as the median (interquartile range).

DM, dermatomyositis; IP, interstitial pneumonia; Dead, dead due to IP; CADM, clinically amyopathic DM; A/SIP, acute/subacute IP; Disease duration, disease duration from onset of respiratory symptoms of IP to initiation of treatments; MDA5, anti-melanoma differentiation-associated gene 5; Ab, antibody; ARS, aminoacyl-tRNA synthetase; CK, creatine kinase; LDH, lactate dehydrogenase; CRP, C-reactive protein; KL-6, Krebs von der Lungen-6; LRG, leucine-rich α2-glycoprotein; AaDO_2_, alveolar-arterial oxygen difference; VC, vital capacity; DLco, diffusion capacity of the lung for carbon monoxide; GGO, ground-glass opacity; PSL, prednisolone; CSA, cyclosporine; TAC, tacrolimus; MPDN pulse, methylprednisolone pulse therapy; IVCY, intravenous pulse cyclophosphamide; IVIg, intravenous high-dose immunoglobulin.

The *P*-values were estimated using Fisher’s exact test or Mann-Whitney U-test.

*P <0.05.

^a^Number of subjects, n = 5.

^b^Number of subjects, n = 9.

^c^Number of subjects, n = 35.

^d^Number of subjects, n = 26.

^e^Number of subjects, n = 15.

^f^Number of subjects, n = 20.

### Serum LRG levels in DM-IP patients

**Fig 1:**
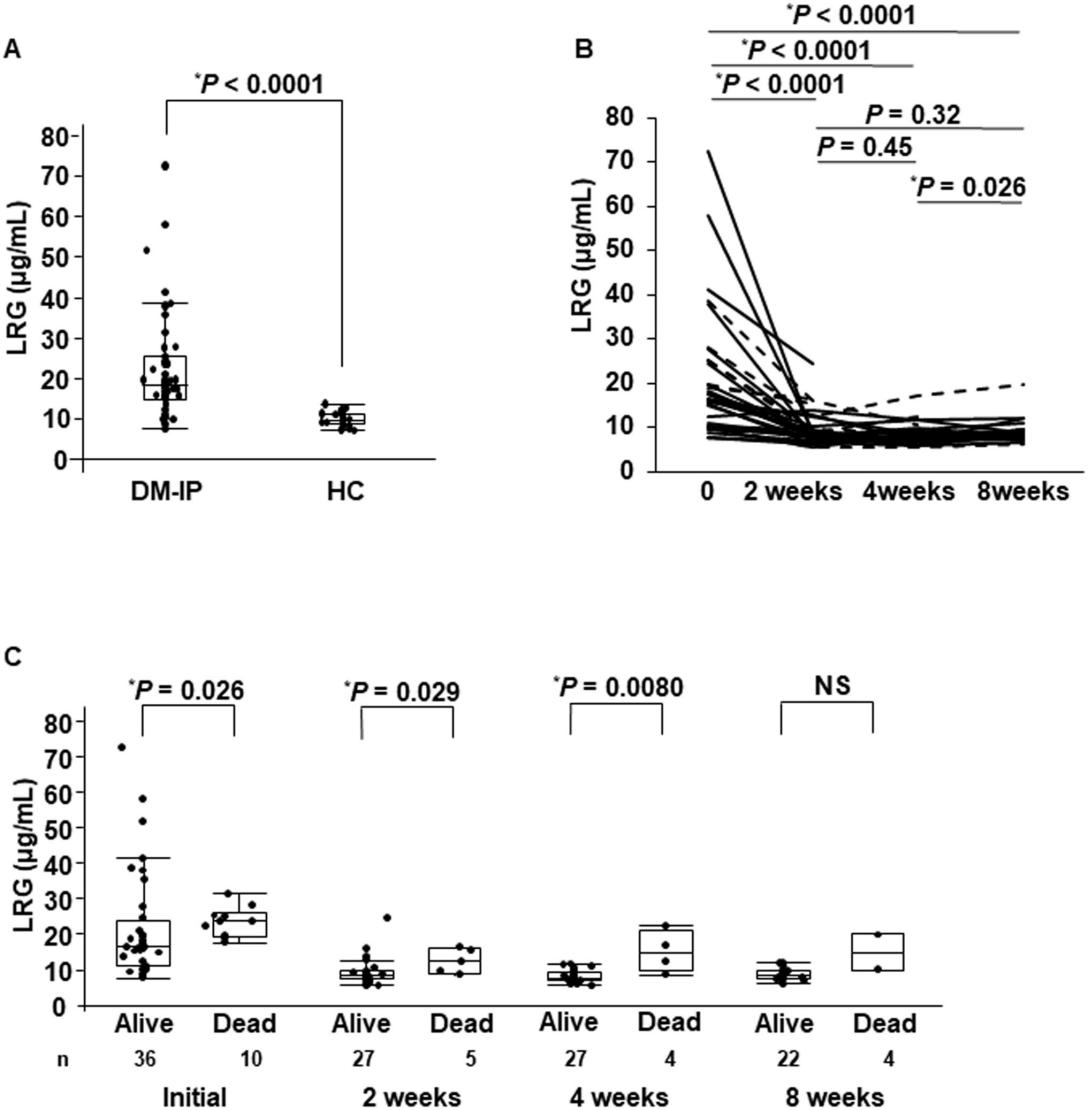
Serum LRG levels in patients with DM-IP and the HC, and changes in serum LRG levels at baseline measurement and at 2, 4, and 8 weeks after the initiation of treatment. The serum LRG levels of the DM-IP patients were higher than those of the HC (A). The serum LRG levels at 2, 4, and 8 weeks after the initiation of treatment were significantly decreased in comparison with those before treatment (B). The serum LRG levels at baseline measurement and 2 and 4 weeks after the initiation of treatment were significantly higher in the patients who died than in the surviving patients. The serum LRG levels at 8 weeks after the initiation of treatment were not significantly higher in the patients who died than in the surviving patients because the number of patients was small (C). LRG, leucine-rich α2-glycoprotein; DM, dermatomyositis; IP, interstitial pneumonia; HC, healthy control subjects; NS, not significant. Dashed line: dead due to IP; solid line: alive.

As shown in Fig 1A, the median levels (interquartile range) of serum LRG were 18.4 (14.6–25.2) in the DM-IP patients (n = 46) and 9.62 (8.65–11.5) μg/mL in the age-matched HC (n = 27). The serum LRG level in the DM-IP patients was higher than that in the HC (*P* < 0.0001). The serum LRG levels were measured at 2, 4, and 8 weeks after treatment initiation in 32, 31, and 24 patients, respectively. As shown in Fig 1B, the serum LRG levels (8.53 [7.57–11.9] μg/mL) at 2 weeks, (8.31 [6.98–9.82] μg/mL) at 4 weeks, and (8.77 [7.69–9.67] μg/mL) at 8 weeks after treatment initiation were significantly decreased compared with those at the initial treatment (18.4 [14.6–25.2] μg/mL) (*P* < 0.0001 for all three).

Serum levels of LRG between the survivors and non-survivors at the baseline measurement and at 2, 4, and 8 weeks after treatment are compared in Fig 1C. The median levels (interquartile range) of serum LRG at these times were significantly higher in the non-survivors (23.7 [19.5–26.0], 12.5 [9.12–15.9], and 14.7 [9.63–20.9] μg/mL, respectively) than in the surviving patients (16.6 [11.3–23.6], 8.32 [7.34–9.58], and 7.71 [6.91–9.42] μg/mL, respectively), (*P* = 0.026, 0.029, and 0.0080, respectively).

### Comparison of disease indicators of DM-IP, including serum LRG levels, on admission between patients with A/SIP and CIP

Table 2 shows the disease indicators of DM-IP on admission of the 33 DM-A/SIP patients and the 13 DM-CIP patients. The anti-MDA5-Ab-positive rate was slightly higher in the DM-A/SIP group than in the DM-CIP group. No significant differences were noted in the anti-ARS-Ab-positive rate or serum CK or KL-6 levels between the two groups. The serum LDH, CRP, and ferritin levels were significantly higher in the DM-A/SIP patients than in the DM-CIP patients (*P* = 0.0010, 0.0021, and 0.0014, respectively). The median level (interquartile range) of serum LRG was significantly higher in the DM-A/SIP patients, 19.7 (16.6–29.6) μg/mL, than in the DM-CIP patients, 10.9 (10.1–17.5) μg/mL (*P* = 0.0004). No significant differences were noted in %VC, %DLco, or total fibrosis score, but the AaDO_2_ and total GGO score were significantly higher in the DM-A/SIP group than in the DM-CIP group (*P* = < 0.0001, 0.0081, respectively).

**Table 2:** Disease indicators and treatment contents of DM-IP on admission of patients with A/SIP and CIP.

Characteristic	A/SIP (n = 33)	CIP (n = 13)	*P*
Positive anti-MDA5-Ab, n (%)	12 (36)	1 (8)	0.073
Positive anti-ARS-Ab, n (%)	12 (36)	8 (62)	0.187
CK, IU/l	132 (59.5–733)	90 (54.5–149)	0.272
LDH, IU/l	352 (257–506)	214 (178–304)	0.0010*
CRP, mg/dl	0.91 (0.14–2.66)	0.12 (0.05–0.17)	0.0021*
KL-6, U/ml	749 (478–2063)	763 (557–1106)	0.903
Ferritin, ng/ml	658 (114–1137)^a^	92.7 (39.1–145)	0.0014*
LRG, μg/ml	19.7 (16.6–29.6)	10.9 (10.1–17.5)	0.0004*
AaDO_2_, mmHg	40.4 (20.9–75.8)	10.5 (3.90–20.3)	< 0.0001*
%VC, %	81.2 (59.5–96.1)^b^	85.3 (77.8–93.7)	0.317
%DLco, %	48.9 (23.6–64.7)^b^	52.5 (42.9–61.2)	0.834
Total GGO score	12.0 (7.7–16.7)	6.9 (6.1–9.7)	0.0081*
Total fibrosis score	3.9 (2.7–5.0)	3.0 (2.0–3.8)	0.118
PSL, mg/day	55 (46–65)^a^	35 (28–48)	0.0002*
CSA, mg/day	238 (219–256)^c^	200 (200–200)^e^	0.284
TAC, mg/day	6 (5.0–8.3)^d^	4.5 (4.0–6.8)^f^	0.0087*
MPDN pulse, n (%)	11 (33)	0 (0)	0.020*
IVCY, n (%)	24 (73)	1 (8)	0.0001*
Total IVCY, mg	1950 (1000–3800)	500 (500–500)	0.164
IVIg, n (%)	11 (33)	0 (0)	0.020*

Laboratory markers are presented as the median (interquartile range).

DM, dermatomyositis; IP, interstitial pneumonia; RPIP, rapid progressive IP; CIP, chronic IP; MDA5, anti-melanoma differentiation-associated gene 5; Ab, antibody; ARS, aminoacyl-tRNA synthetase; CK, creatine kinase; LDH, lactate dehydrogenase; CRP, C-reactive protein; KL-6, Krebs von der Lungen-6; LRG, leucine-rich α2-glycoprotein; AaDO_2_, alveolar-arterial oxygen difference; VC, vital capacity; DLco, diffusion capacity of the lung for carbon monoxide; GGO, ground-glass opacity; PSL, prednisolone; CSA, cyclosporine; TAC, tacrolimus; MPDN pulse, methylprednisolone pulse therapy; IVCY, intravenous pulse cyclophosphamide; IVIg, intravenous high-dose immunoglobulin.

The *P*-values were estimated using Fisher’s exact test or Mann-Whitney U-test.

*P <0.05.

^a^Number of subjects, n = 32.

^b^Number of subjects, n = 18.

^c^Number of subjects, n = 24.

^d^Number of subjects, n = 9.

^e^Number of subjects, n = 1.

^f^Number of subjects, n = 11.

### Comparison of disease indicators of DM-IP, including serum LRG levels, on admission between patients positive for anti-MDA5-Ab or for anti-ARS-Ab

S1 Table shows the disease indicators of DM-IP on admission of the 13 anti-MDA5-Ab-positive DM-IP patients and the 20 anti-ARS-Ab-positive DM-IP patients. The percentage of patients with A/SIP, and the serum LDH and CRP levels, were slightly higher in the anti-MDA5-Ab-positive group than in the anti-ARS-Ab-positive group. The serum ferritin levels and AaDO_2_ were also significantly higher in the anti-MDA5-Ab-positive group than in the anti-ARS-Ab-positive group (*P* = 0.0017 and 0.0024, respectively). The median level (interquartile range) of serum LRG was slightly higher in the anti-MDA5-Ab-positive group, 21.0 (18.3–25.2) μg/mL, than in the anti-ARS-Ab-positive group, 15.9 (10.2–25.7) μg/mL (*P* = 0.0801). No significant differences were noted in the other indicators.

### Correlation between serum LRG levels and other disease activity indicators of DM-IP

As shown in Table 3, the serum LRG levels correlated significantly with the serum levels of LDH (*R* = 0.467, *P* = 0.0011), CRP (*R* = 0.710, *P* < 0.0001), ferritin (*R* = 0.546, *P* = 0.0001), AaDO_2_ (*R* = 0.559, *P* < 0.0001), %DLco (*R* = -0.383, *P* = 0.037), and total GGO score (*R* = 0.392, *P* = 0.0071). There was no correlation between the serum LRG levels and CK, KL-6, %VC, or total fibrosis score.

**Table 3:** Correlation between serum LRG levels and other disease activity indicators of DM-IP.

Variables	Correlation *R*	Analysis *P*
CK	-0.084	0.580
LDH	0.467	0.0011*
CRP	0.710	< 0.0001*
KL-6	-0.035	0.816
Ferritin	0.546	0.0001*
AaDO_2_	0.559	< 0.0001*
%VC	-0.041	0.825
%DLco	-0.383	0.037*
Total GGO score	0.392	0.0071*
Total fibrosis score	0.208	0.165

LRG, leucine-rich α2-glycoprotein; DM, dermatomyositis; IP, interstitial pneumonia; CK, creatine kinase; LDH, lactate dehydrogenase; CRP, C-reactive protein; KL-6, Krebs von der Lungen-6; AaDO_2_, alveolar-arterial oxygen difference; VC, vital capacity; DLco, diffusion capacity of the lung for carbon monoxide; GGO, ground-glass opacity.

*R*, correlation coefficient established using Spearman correlation coefficients.

*P<0.05.

### Cut-off values of serum LRG levels for determining prognosis, progression of IP, and anti-MDA5-Ab positivity in DM-IP patients

To clarify the cut-off point effective for determining IP progression and anti-MDA5-Ab positivity in DM-IP patients, ROC curve analysis was performed using the initial serum LRG levels. The value that maximized the area under the ROC curve was 15.7 μg/mL for A/SIP (sensitivity: 90.9%, specificity: 76.9%) and 17.6 μg/mL for anti-MDA5-Ab positivity (sensitivity: 84.6%, specificity: 57.6%). To clarify the cut-off point effective for determining poor prognosis, ROC curve analysis was performed using the serum LRG levels measured initially and at 2 weeks and 4 weeks after treatment initiation. The value that maximized the area under the ROC curve was 17.6 μg/mL for the initial serum LRG level (sensitivity: 100%, specificity: 58.3%), 8.7 μg/mL for the serum LRG level at 2 weeks (sensitivity: 100%, specificity: 63.0%), and 12.5 μg/mL for the serum LRG level at 4 weeks after treatment initiation (sensitivity: 75.0%, specificity: 100%).

**Fig 2:**
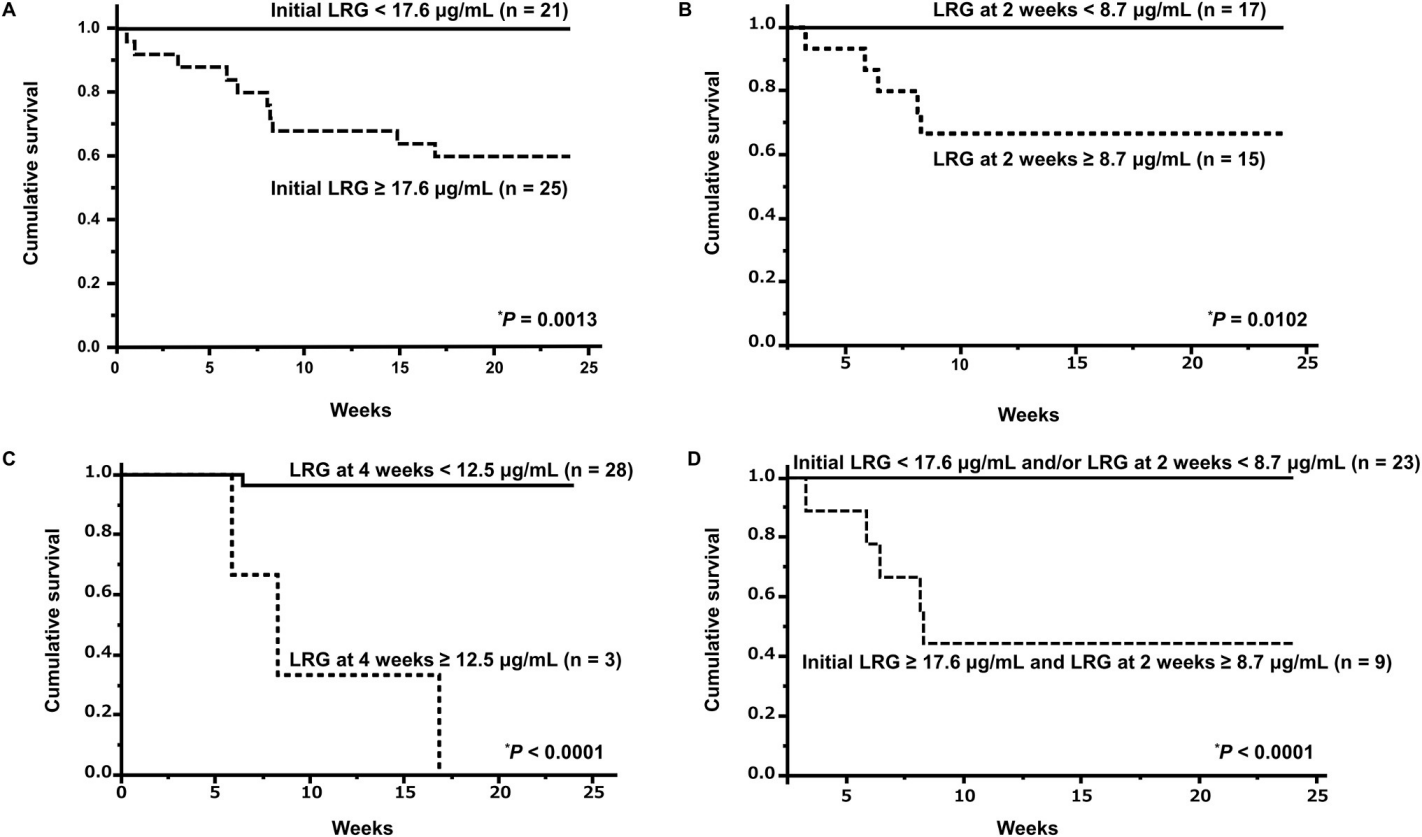
Survival curves of patients with DM-IP based on their serum LRG levels. The survival rate after 24 weeks in patients with an initial LRG level ≥ 17.6 μg/mL (survival rate: 60%) was significantly lower than that in patients with an initial LRG level < 17.6 μg/mL (100%) (*P* = 0.0013). Solid line: < 17.6 μg/mL, dashed line: ≥ 17.6 μg/mL (A). The survival rate after 24 weeks in patients with LRG level at 2 weeks after treatment ≥ 8.7 μg/mL (67%) was significantly lower than that in patients with LRG level at 2 weeks after treatment < 8.7 μg/mL (100%) (*P* = 0.0149). Solid line: < 8.7 μg/mL, dashed line: ≥ 8.7 μg/mL (B). The survival rate after 24 weeks in patients with LRG level at 4 weeks after treatment ≥ 12.5 μg/mL (0%) was significantly lower than that in patients with LRG level at 4 weeks after treatment < 12.5 μg/mL (96.8%) (*P* = 0.0009). Solid line: < 12.5 μg/mL, dashed line: ≥ 12.5 μg/mL (C). The survival rate after 24 weeks in patients with an initial LRG level ≥ 17.6 μg/mL and that at 2 weeks ≥ 8.7 μg/mL (survival rate: 44%) was significantly lower than that in patients with an initial LRG level < 17.6 μg/mL and/or that at 2 weeks < 8.7 μg/mL (100%) (*P* < 0.0001). Solid line: an initial LRG level < 17.6 μg/mL and/or that at 2 weeks < 8.7 μg/mL, dashed line: an initial LRG level ≥ 17.6 μg/mL and that at 2 weeks ≥ 8.7 μg/mL (D). Survival rates were calculated by the Kaplan-Meier method and compared by a log-rank test. *P < 0.05. DM, dermatomyositis; IP, interstitial pneumonia; LRG, leucine-rich α2-glycoprotein.

The ratios of A/SIP and positive anti-MDA5-Ab by the higher or lower of the cut-off values of initial serum LRG level, and patients dead due to IP by the higher or lower of the cut-off values of initial, 2-week, and 4-week serum LRG levels are shown in Table 4. The rate of A/SIP was significantly higher in the patients with an initial serum level of LRG ≥ 15.7 pg/mL (survival rate: 91%) than in those with < 15.7 pg/mL (23%) (*P* < 0.0001). The rate of anti-MDA5-Ab positivity was significantly higher in the patients with an initial serum level of LRG ≥ 17.6 pg/mL (44%) than in those with < 17.6 pg/mL (10%) (*P* = 0.0194). The survival rate after 24 weeks was significantly lower in the patients with an initial serum level of LRG ≥ 17.6 pg/mL (40%) than in those with < 17.6 pg/mL (100%) (*P* = 0.0009). The survival rate after 24 weeks was significantly lower in the patients with a serum level of LRG at 2 weeks after treatment ≥ 8.7 pg/mL (67%) than in those with < 8.7 pg/mL (100%) (*P* = 0.0149). The survival rate after 24 weeks was significantly lower in the patients with a serum level of LRG at 4 weeks after treatment ≥ 12.5 pg/mL (0%) than in those with < 12.5 pg/mL (96.8%) (*P* = 0.0009). The patients were then divided into two groups based on cut-off values for prognosis, and Kaplan-Meier survival curves were plotted (Fig 2A–2C).

**Table 4:** Ratios of A/SIP and positive anti-MDA5-Ab by the higher or lower of the cut-off values of initial serum LRG level, and patients dead due to IP by the higher or lower of the cut-off values of initial, 2-week, and 4-week serum LRG levels.

Characteristic	Cutoff value of LRG (μg/ml)		*P*	AUC	Se (%)	Sp (%)	PPV	NPV	Odds ratio (95% CI)
A/SIP, n (%)	Initial LRG ≥ 15.7 (n = 33)	Initial LRG < 15.7 (n = 13)							
	30 (91)	3 (23)	< 0.0001*	0.839	90.9	76.9	90.9	76.9	33.3 (5.77–192.4)
	Initial LRG ≥ 17.6 (n = 25)	Initial LRG < 17.6 (n = 21)							
Positive anti-MDA5-Ab, n (%)	11 (44)	2 (10)	0.0194*	0.646	84.6	57.6	44.0	90.5	7.46 (1.42–39.1)
	Initial LRG ≥ 17.6 (n = 25)	Initial LRG < 17.6 (n = 21)							
Dead, n (%)	10 (40)	0 (0)	0.0009*	0.733	100	58.3	40.0	100	NC
	LRG at 2 weeks ≥ 8.7 (n = 15)	LRG at 2 weeks < 8.7 (n = 17)							
Dead, n (%)	5 (33)	0 (0)	0.0149*	0.815	100	63.0	33.3	100	NC
	LRG at 4 weeks ≥ 12.5 (n = 3)	LRG at 4 weeks < 12.5 (n = 28)							
Dead, n (%)	3 (100)	1 (3.2)	0.0009*	0.917	75.0	100	100	96.4	NC

IP, interstitial pneumonia; A/SIP, acute/subacute IP; MDA5, anti-melanoma differentiation-associated gene 5; Ab, antibody; LRG, leucine-rich α2-glycoprotein; Dead, dead due to IP; Se, sensitivity; Sp, specificity; PPV, positive predictive value; NPV, negative predictive value; AUC, area under the curve; CI, confidence interval; NC, not calculated because the data were sparse. The *P*-values were estimated using Fisher’s exact test.

*P <0.05.

The patients were divided into the following two groups: patients with initial level of LRG < 17.6 μg/mL and/or that at 2 weeks < 8.7 μg/mL and patients with initial level of LRG ≥ 17.6 μg/mL and that at 2 weeks ≥ 8.7 μg/mL, according to the cut-off value predicting prognosis. The survival rate after 24 weeks was significantly lower in the patients with an initial LRG level ≥ 17.6 μg/mL and that at 2 weeks ≥ 8.7 μg/mL (survival rate: 44%) than in those with an initial LRG level < 17.6 μg/mL and/or that at 2 weeks < 8.7 μg/mL (100%) (*P* < 0.0001) (Fig 2D).

### Cut-off values of initial serum CRP, KL-6, and ferritin levels and AaDO_2_ level for determining prognosis in DM-IP patients

To clarify the cut-off point effective for determining poor prognosis, ROC curve analysis was performed using the initial serum CRP, KL-6, and ferritin levels and AaDO_2_ level. The value that maximized the area under the ROC curve was 2.9 mg/dl for the initial serum CRP level (sensitivity: 10.0%, specificity: 80.6%), 1047 U/ml for the serum KL-6 level (sensitivity: 60.0%, specificity: 72.2%), 1005 ng/ml for the serum ferritin level (sensitivity: 80.0%, specificity: 85.7%), and 35.6 mmHg for the AaDO_2_ level (sensitivity: 100%, specificity: 77.8%).

The ratios of patients dead due to IP by the higher or lower of the cut-off values of initial serum CRP, KL-6, and ferritin levels and AaDO_2_ level are shown in S2 Table. The survival rate after 24 weeks was significantly lower in the patients with an initial serum level of ferritin ≥ 1005 ng/ml (survival rate: 38%) than in those with < 1005 ng/ml (93.7%) (*P* = 0.0002). The survival rate after 24 weeks was significantly lower in the patients with an initial level of AaDO_2_ ≥ 35.6 mmHg (44%) than in those with < 35.6 mmHg (100%) (*P* < 0.0001). The survival rate after 24 weeks was not significantly lower in the patients with an initial serum level of CRP ≥ 2.9 mg/dl (87%) than in those with < 2.9 mg/dl (76%) (*P* = 0.6641) or in the patients with an initial serum level of KL-6 ≥ 1047 U/ml l (62%) than in those with < 1047 U/ml (87%) (*P* = 0.0741). The patients were then divided into two groups based on cut-off values for prognosis, and Kaplan-Meier survival curves were plotted (S1 Fig).

Univariate analysis results revealed that the levels of initial serum LRG ≥ 17.6 μg/mL, ferritin ≥ 1005 ng/mL, and AaDO_2_ ≥ 35.6 mmHg were associated with death due to IP. Multivariate analysis using Cox proportional hazards regression analysis results revealed that the levels of initial serum LRG ≥ 17.6 μg/mL and AaDO_2_ ≥ 35.6 mmHg were independently associated with death due to IP (*P* = 0.031 and 0.0015, respectively), whereas serum ferritin ≥ 1005 ng/mL was not significant factor in multivariate analysis (*P* = 0.21).

Initial serum prognostic biomarkers of DM-IP such as LRG, KL-6, and ferritin levels were examined using Cox proportional hazards regression analysis, but no significant differences were obtained due to the small sample size.

### Survival rates using a combination of initial serum LRG and ferritin levels, and AaDO_2_ level in DM-IP patients

**Fig 3:**
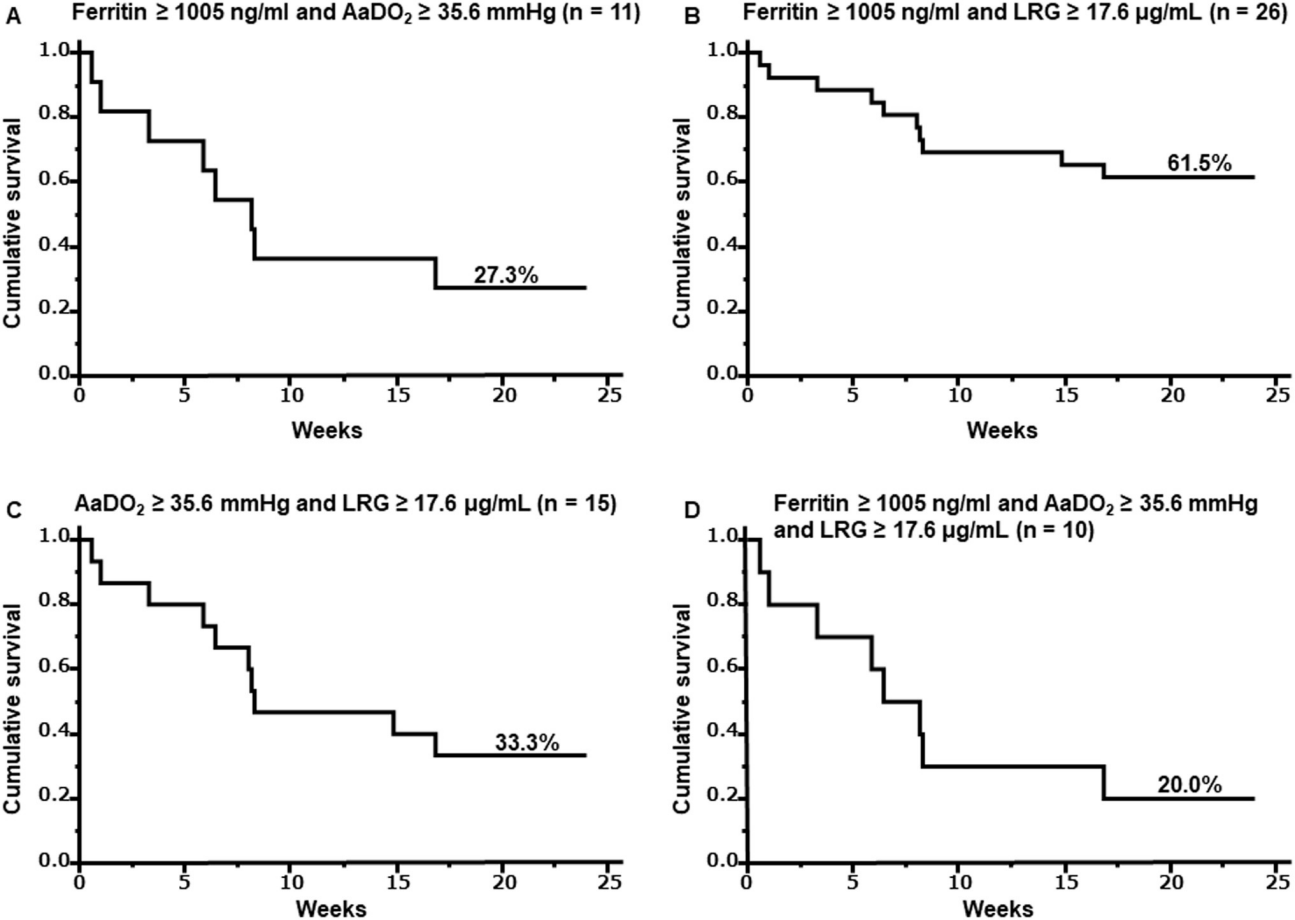
Survival curves using a combination of initial serum LRG and ferritin levels, and AaDO_2_ level in DM-IP patients. The survival rate after 24 weeks was 27.3% for the patients with an initial serum level of ferritin ≥ 1005 ng/ml and AaDO_2_ ≥ 35.6 mmHg. Solid line: Ferritin ≥ 1005 ng/ml and AaDO_2_ ≥ 35.6 mmHg (A). The survival rate after 24 weeks was 61.5% for the patients with an initial serum level of ferritin ≥ 1005 ng/ml and LRG ≥ 17.6 μg/mL. Solid line: Ferritin ≥ 1005 ng/ml and LRG ≥ 17.6 μg/mL (B). The survival rate after 24 weeks was 33.3% for the patients with an initial AaDO_2_ ≥ 35.6 mmHg and serum level of LRG ≥ 17.6 μg/mL. Solid line: AaDO_2_ ≥ 35.6 mmHg and LRG ≥ 17.6 μg/mL (C). The survival rate after 24 weeks was 20.0% for the patients with an initial serum level of ferritin ≥ 1005 ng/ml, AaDO_2_ ≥ 35.6 mmHg, and initial serum level of LRG ≥17.6 μg/mL. Solid line: Ferritin ≥ 1005 ng/ml and AaDO_2_ ≥ 35.6 mmHg and LRG ≥ 17.6 μg/mL (D). Survival rates were calculated by the Kaplan-Meier method and compared by a log-rank test. *P < 0.05. DM, dermatomyositis; IP, interstitial pneumonia; LRG, leucine-rich α2-glycoprotein; AaDO_2_, alveolar-arterial oxygen difference.

The survival rate after 24 weeks was 27.3% for the patients with an initial serum level of ferritin ≥ 1005 ng/ml and AaDO_2_ ≥ 35.6 mmHg, 61.5% for the patients with an initial serum level of ferritin ≥ 1005 ng/ml and LRG ≥ 17.6 μg/mL, 33.3% for the patients with an initial AaDO_2_ ≥ 35.6 mmHg and serum level of LRG ≥ 17.6 μg/mL, and 20.0% for the patients with an initial serum level of ferritin ≥ 1005 ng/ml, AaDO_2_ ≥ 35.6 mmHg, and initial serum level of LRG ≥ 17.6 μg/mL (Fig 3).

## Discussion

Among the DM-IP patients in this study, the anti-MDA5 Ab-positive rate, the serum ferritin level at the initiation of treatment, the AaDO_2_ level, and the GGO score on chest HRCT were significantly higher in the fatal IP group than in the survivor group, which was consistent with previous reports. The serum LRG levels of these DM-IP patients were significantly higher than those of the HC. In addition, the initial serum LRG levels in the A/SIP groups were significantly higher than those in the CIP groups. Furthermore, the serum LRG level in the fatal IP group was significantly higher than that in the survivor group at the initiation of treatment and at 2 and 4 weeks thereafter. Compared to single point LRG levels, combining the levels of initial serum LRG and those at 2 weeks after the initiation of treatment resulted in a large difference in survival rate, suggesting that they may be more useful for predicting prognosis. These results suggest that LRG may be a useful marker of disease activity in DM-IP. In accordance with this, the initial serum LRG level correlated significantly with the indicators of disease activity and prognosis in DM-IP (serum LDH and ferritin levels, AaDO_2_ level, and total GGO score), suggesting that it has the potential to be used as a substitute for these indices.

In a murine model of lung fibrosis, LRG was upregulated in the alveolar epithelial cells, bronchial epithelial cells, endothelial cells, and infiltrated immune cells [23]. As suggested by a study on inflammatory bowel disease [24], local induction of LRG may be mediated by various cytokines. High levels of serum IL-6, IL-8, IL-10, IL-18, CCL2, TNF-α, IFN-α, and IP-10 were detected in PM/DM-IP cases, and activated macrophages, Th1-type T lymphocytes, and neutrophils are thought to be involved in the pathogenesis [25–29]. Because serum IL-8, IL-6, CCL2, and IL-10 levels are high in anti-MDA5 Ab-positive patients and in patients with poor prognosis, activated macrophages and neutrophils are considered to be related to the severity of DM-IP [25, 29]. In the pulmonary lesions of DM-IP, infiltrated immune cells and their production of these cytokines, which are candidate indicators of the disease activity of DM-IP, may contribute to the upregulation of LRG. Thus, local LRG production during active disease may be involved in the elevation of serum LRG and may help to reflect the progression, disease activity, and outcome of DM-IP.

High levels of initial serum ferritin, CRP, KL-6, and AaDO_2_ have been reported as poor prognostic markers by blood testing in DM-IP patients other than those who are anti-MDA5 Ab-positive [6, 30, 31]. In the present study, initial serum ferritin and AaDO_2_ levels were significantly higher in the death group than in the surviving group, which supports the previous reports. Further, multivariate analysis results revealed that elevated AaDO_2_ and elevated serum LRG levels independently contributed significantly to DM-IP mortality, but no significant difference was observed in elevated serum ferritin levels. AaDO_2_ measured using arterial blood gas analysis reflects the respiratory status and is useful for predicting the prognosis of DM-IP; however, serum LRG levels may also support further prognostic predictions as a serological biomarker.

Attempts have been made to extract poor prognostic cases by combining multiple poor prognostic factors in DM-IP patients. Patients with both high initial serum ferritin and AaDO_2_ levels are reported to have a poorer prognosis than those with high levels of one or the other alone [31]. In the present study, we examined initial serum LRG levels in addition to initial serum ferritin levels and AaDO_2_ levels and suggest that they may be more useful in extracting cases with poor prognosis than when each is used alone.

LRG functions to modulate TGF-β signal transmission [32, 33] and was shown to enhance TGF-β signaling in fibroblasts and promote fibrosis in a pulmonary fibrosis model [34]. However, in the present study, the serum LRG level before treatment did not correlate with the fibrosis score. During the acute phase of DM-IP, pulmonary inflammation—rather than fibrosis—is the main pathology. In DM-IP, GGO and consolidation are the main findings on chest HRCT, whereas honeycombing, which is an indicator of fibrosis, is not observed [35]. Thus, the association between LRG and fibrosis should be evaluated in patients in the chronic phase of DM-IP or in patients with other IPs, such as idiopathic pulmonary fibrosis. Additional studies are necessary to evaluate the association between the serum LRG level and pulmonary fibrosis.

## Conclusions

In conclusion, we propose the serum LRG level as a promising biomarker of disease progression, activity, and prognosis in patients with DM-IP. The present study had some limitations. In particular, it was a retrospective study of a relatively small number of patients that was performed at a single institution. The investigation of additional cases in multiple institutions will be required to clarify the usefulness of LRG as an indicator of the disease progression, activity, and prognosis of DM-IP; thus, further studies are needed.

## Supporting information

S1 Table Comparison of the disease indicators of DM-IP on admission between patients with positive anti-MDA5-Ab and anti-ARS-Ab. The laboratory markers are presented as the median (interquartile range). DM, dermatomyositis; IP, interstitial pneumonia; MDA5, anti-melanoma differentiation-associated gene 5; Ab, antibody; ARS, aminoacyl-tRNA synthetase; CADM, clinically amyopathic DM; A/SIP, acute/subacute IP; CK, creatine kinase; LDH, lactate dehydrogenase; CRP, C-reactive protein; LRG, leucine-rich α2 glycoprotein; AaDO_2_, alveolar-arterial oxygen difference; VC, vital capacity; DLco, diffusion capacity of the lung for carbon monoxide; GGO, ground-glass opacity. The *P*-values were estimated using Fisher’s exact test or Mann-Whitney U- test. *P < 0.05. ^a^Number of subjects, n = 19. ^b^Number of subjects, n = 14.
(DOCX)

S2 Table Ratios of patients dead due to IP by the higher or lower of the cut-off values of initial serum CRP, KL-6, and ferritin levels and AaDO_2_ level. IP, interstitial pneumonia; CRP, C-reactive protein; KL-6, Krebs von der Lungen-6; AaDO_2_, alveolar-arterial oxygen difference; Dead, dead due to IP; Se, sensitivity; Sp, specificity; PPV, positive predictive value; NPV, negative predictive value; AUC, area under the curve; CI, confidence interval; NC, not calculated because the data were sparse. The *P*-values were estimated using Fisher’s exact test. *P < 0.05.
(DOCX)

S1 Fig Survival curves of patients with DM-IP based on their initial serum CRP, KL-6, and ferritin levels and AaDO_2_ level. The survival rate after 24 weeks in patients with an initial serum level of CRP ≥ 2.9 mg/dl (survival rate: 87%) versus those with < 2.9 mg/dl (76%) (*P* = 0.6641). Solid line: < 2.9 mg/dl, dashed line: ≥ 2.9 mg/dl (A). The survival rate after 24 weeks in patients with an initial serum level of KL-6 ≥ 1047 U/ml (38%) versus those with < 1047 U/ml (87%) (*P* = 0.0741). Solid line: < 1047 U/ml, dashed line: ≥ 1047 U/ml (B). The survival rate after 24 weeks in patients with an initial serum level of ferritin ≥ 1005 ng/ml (38%) versus those with < 1005 ng/ml (93.7%) (*P* = 0.0002). Solid line: < 1005 ng/ml, dashed line: ≥ 1005 ng/ml (C). The survival rate after 24 weeks in patients with an initial serum level of AaDO_2_ ≥ 35.6 mmHg (44%) versus those with < 35.6 mmHg (100%) (*P* < 0.0001). Solid line: < 35.6 mmHg, dashed line: ≥ 35.6 mmHg (D). Survival rates were calculated by the Kaplan-Meier method and compared by a log-rank test. *P < 0.05. DM, dermatomyositis; IP, interstitial pneumonia; CRP, C-reactive protein; KL-6, Krebs von der Lungen-6; AaDO_2_, alveolar-arterial oxygen difference.
(TIF)
