# Supplementary material for: Correlation of increased serum leucine-rich α2-glycoprotein levels with disease prognosis, progression, and activity of interstitial pneumonia in patients with dermatomyositis: A retrospective study
Source: PLoS One. 2020 Jun 1;15(6):e0234090. doi: 10.1371/journal.pone.0234090 (PMC7263588; doi:10.1371/journal.pone.0234090)
Supplement: S1 Table — The laboratory markers are presented as the median (interquartile range). DM, dermatomyositis; IP, interstitial pneumonia; MDA5, anti-melanoma differentiation-associated gene 5; Ab, antibody; ARS, aminoacyl-tRNA synthetase; CADM, clinically amyopathic DM; A/SIP, acute/subacute IP; CK, creatine kinase; LDH, lactate dehydrogenase; CRP, C-reactive protein; LRG, leucine-rich α2 glycoprotein; AaDO2, alveolar-arterial oxygen difference; VC, vital capacity; DLco, diffusion capacity of the lung for carbon monoxide; GGO, ground-glass opacity. The P-values were estimated using Fisher’s exact test or Mann-Whitney U- test. *P < 0.05. aNumber of subjects, n = 19. bNumber of subjects, n = 14. (DOCX) [file pone.0234090.s001.docx]

| **Supplementary table 1. Comparison of the disease indicators of DM-IP on admission between patients with positive anti-MDA5-Ab and anti-ARS-Ab** | | | |
| --- | --- | --- | --- |
| Characteristics | anti-ARS-Ab positive (n =20) | anti-MDA5-Ab positive (n =13) | *P* |
| CADM, n (%) | 16 (80) | 10 (77) | 1.000 |
| A/SIP, n (%) | 12 (60) | 12 (92) | 0.056 |
| CK, IU/l | 91.5 (62.0-158) | 115 (60-524) | 0.768 |
| LDH, IU/l | 257 (190-380) | 336 (318-494) | 0.053 |
| CRP, mg/dl | 0.16 (0.05-1.78) | 0.93 (0.59-2.13) | 0.090 |
| KL-6, U/ml | 938 (599-2320) | 672 (451-1808) | 0.348 |
| Ferritin, ng/ml | 151 (57.1-619) ^a^ | 1108 (610-1936) | 0.0017* |
| LRG, μg/ml | 15.9 (10.2-25.7) | 21.0 (18.3-25.2) | 0.0801 |
| AaDO_2_, mmHg | 18.5 (5.6-39.0) | 52.5 (38.7-94.4) | 0.0024* |
| %VC, % | 84.2 (73.2-91.2) ^b^ | 85.6 (50.6-104.9) | 0.902 |
| %DLco, % | 52.4 (32.0-61.2) ^b^ | 29.9 (14.4-63.2) | 0.273 |
| Total GGO score | 10.7 (7.1-14.8) | 12.0 (5.8-17.7) | 0.593 |
| Total fibrosis score | 3.6 (2.9-4.9) | 5.0 (2.2-5.0) | 0.475 |
| The laboratory markers are presented as the median (interquartle range). DM, dermatomyositis. IP, interstitial pneumonia. MDA5, anti-melanoma differentiation-associated gene 5. Ab, antibody. ARS, aminoacyl-tRNA synthetase. CADM, clinically amyopathic DM. A/SIP, acute/subacute IP. CK, creatine kinase. LDH, lactate dehydrogenase. CRP, C-reactive protein. LRG, leucine rich α2 glycoprotein. AaDO_2_, alveolar-arterial oxygen difference. VC, vital capacity. DLco, diffusion capacity of the lung for carbon monoxide. GGO, ground-glass opacity. The P-values were estimated using Fisher’s exact test or Mann-Whitney U- test. **P* <0.05. ^a^Number of subjects, n= 19. ^b^Number of subjects, n= 14. | | | |
