# Supplementary material for: Correlation of increased serum leucine-rich α2-glycoprotein levels with disease prognosis, progression, and activity of interstitial pneumonia in patients with dermatomyositis: A retrospective study
Source: PLoS One. 2020 Jun 1;15(6):e0234090. doi: 10.1371/journal.pone.0234090 (PMC7263588; doi:10.1371/journal.pone.0234090)
Supplement: S2 Table — IP, interstitial pneumonia; CRP, C-reactive protein; KL-6, Krebs von der Lungen-6; AaDO2, alveolar-arterial oxygen difference; Dead, dead due to IP; Se, sensitivity; Sp, specificity; PPV, positive predictive value; NPV, negative predictive value; AUC, area under the curve; CI, confidence interval; NC, not calculated because the data were sparse. The P-values were estimated using Fisher’s exact test. *P < 0.05. (DOCX) [file pone.0234090.s002.docx]

**Supplementary table 2. Ratios of patients dead due to IP by the higher or lower of cutoff values of initial serum CRP, KL-6, and ferritin levels and AaDO_2_ levels**

| Characteristic | Cutoff value | | *P* | AUC | Se % | Sp % | PPV | NPV | Odds ratio (95%CI) |
| --- | --- | --- | --- | --- | --- | --- | --- | --- | --- |
|  | CRP ≥ 2.9 mg/dl (n = 8) | CRP < 2.9 mg/dl (n = 38) |  |  |  |  |  |  |  |
| Dead, n (%) | 1 (13) | 9 (24) | 0.6641 | 0.308 | 10.0 | 80.6 | 12.5 | 76.3 | 0.46 (0.0498 - 4.26) |
|  |  |  |  |  |  |  |  |  |  |
|  | KL-6 ≥ 1047 U/ml (n = 16) | KL-6 < 1047 U/ml (n = 30) |  |  |  |  |  |  |  |
| Dead, n (%) | 6 (38) | 4 (13) | 0.0741 | 0.661 | 60.0 | 72.2 | 37.5 | 86.7 | 3.9 (0.91 - 16.8) |
|  |  |  |  |  |  |  |  |  |  |
|  | Ferritin ≥ 1005 ng/ml (n = 13) | Ferritin < 1005 ng/ml (n = 32) |  |  |  |  |  |  |  |
| Dead, n (%) | 8 (62) | 2 (6.3) | 0.0002* | 0.851 | 80.0 | 85.7 | 61.5 | 93.8 | 24 (3.9 - 147.5) |
|  |  |  |  |  |  |  |  |  |  |
|  | AaDO_2_ ≥ 35.6 mmHg (n = 18) | AaDO_2_ < 35.6 mmHg (n = 28) |  |  |  |  |  |  |  |
| Dead, n (%) | 10 (56) | 0 (0) | <0.0001* | 0.900 | 100 | 77.8 | 55.6 | 100 | NC |

IP; interstitial pneumonia, CRP; C-reactive protein, KL-6, Krebs von der Lungen-6; AaDO_2_, alveolar-arterial oxygen difference, Dead; dead due to IP, Se; sensitivity, Sp; specificity, PPV: positive predictive value, NPV; negative predictive value, AUC, area under the curve; CI; confidence interval, NC; not calculated because the datas were sparse. The *P*-values were estimated using Fisher’s exact test. **P* <0.05.
