# Supplementary figures and images for: Correlation of increased serum leucine-rich α2-glycoprotein levels with disease prognosis, progression, and activity of interstitial pneumonia in patients with dermatomyositis: A retrospective study
Source: PLoS One. 2020 Jun 1;15(6):e0234090. doi: 10.1371/journal.pone.0234090 (PMC7263588; doi:10.1371/journal.pone.0234090)

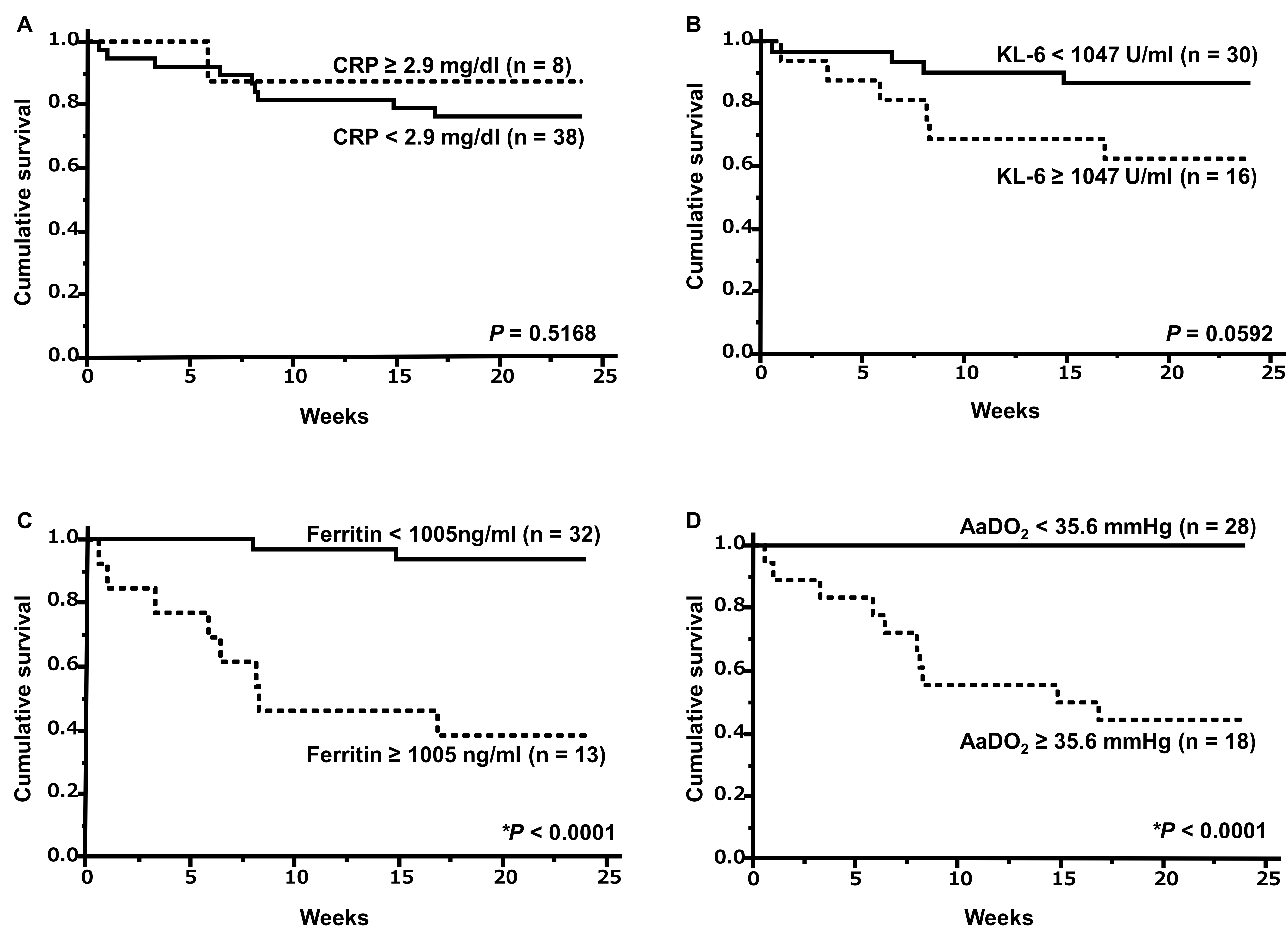

Supplement: S1 Fig — The survival rate after 24 weeks in patients with an initial serum level of CRP ≥ 2.9 mg/dl (survival rate: 87%) versus those with < 2.9 mg/dl (76%) (P = 0.6641). Solid line: < 2.9 mg/dl, dashed line: ≥ 2.9 mg/dl (A). The survival rate after 24 weeks in patients with an initial serum level of KL-6 ≥ 1047 U/ml (38%) versus those with < 1047 U/ml (87%) (P = 0.0741). Solid line: < 1047 U/ml, dashed line: ≥ 1047 U/ml (B). The survival rate after 24 weeks in patients with an initial serum level of ferritin ≥ 1005 ng/ml (38%) versus those with < 1005 ng/ml (93.7%) (P = 0.0002). Solid line: < 1005 ng/ml, dashed line: ≥ 1005 ng/ml (C). The survival rate after 24 weeks in patients with an initial serum level of AaDO2 ≥ 35.6 mmHg (44%) versus those with < 35.6 mmHg (100%) (P < 0.0001). Solid line: < 35.6 mmHg, dashed line: ≥ 35.6 mmHg (D). Survival rates were calculated by the Kaplan-Meier method and compared by a log-rank test. *P < 0.05. DM, dermatomyositis; IP, interstitial pneumonia; CRP, C-reactive protein; KL-6, Krebs von der Lungen-6; AaDO2, alveolar-arterial oxygen difference. (TIF) [file pone.0234090.s003.tif]
